# Veronika Settele 2020: *Revolution im Stall. Landwirtschaftliche Tierhaltung in Deutschland* und Paul R. Josephson 2020: *Chicken. A History from Farmyard to Factory*

**DOI:** 10.1007/s00048-022-00344-9

**Published:** 2022-08-25

**Authors:** Ulrike Heitholt

**Affiliations:** Universität Kassel, Kassel, Deutschland

Veronika Settele 2020: *Revolution im Stall. Landwirtschaftliche Tierhaltung in Deutschland 1945–1990. *Göttingen: Vandenhoeck & Ruprecht, geb., 394 S., 36 Abb., 65,00 €, ISBN 978-3-525-31122‑6.

Paul R. Josephson 2020: *Chicken. A History from Farmyard to Factory. *Cambridge, UK: Polity Press, geb., 252 S., zahlr. Abb., 25,00 US$, ISBN 978-1509525911.

Landwirtschaftlich genutzte Tiere spielten in der geschichtswissenschaftlichen Forschung bisher meist eine Nebenrolle. Sie traten vor allem als Wirtschaftsobjekte in der Agrargeschichte in Erscheinung, oder ihr Verschwinden aus dem öffentlichen Raum diente der Tiergeschichte als Indikator für den Übergang in die Moderne. In jüngerer Zeit aber rücken die landwirtschaftlichen Tiere immer öfter in den direkten Fokus der historischen Forschung – eine begrüßenswerte Entwicklung gerade vor dem Hintergrund aktueller Debatten um Tierwohl, Haltungspraktiken und Klimaschutz. Veronika Settele und Paul R. Josephson tragen mit ihren jüngst erschienenen Monographien dazu bei.

Settele betrachtet die sich wandelnden Praktiken im Umgang von Mensch und Tier in den landwirtschaftlichen Ställen in Deutschland in der Zeit nach dem Zweiten Weltkrieg und begreift diese Entwicklung als „Revolution“: aufgrund der enormen Produktivitätssteigerungen und der „kulturelle[n] Neuausrichtung des Verhältnisses zwischen der Gesellschaft und ihren zur Lebensmittelproduktion gehaltenen Tieren“ (20). Die Autorin untersucht das Geschehen in beiden deutschen Staaten in der Zusammenschau, nicht in der Gegenüberstellung. Das ermöglicht ihr, jenseits politischer Ideologien Erklärungen zu finden für ihren „kontraintuitiven Befund“ (14), dass 1990 die Ähnlichkeiten in der landwirtschaftlichen Tierhaltung trotz jahrzehntelanger Systemkonkurrenz dominierten.

Sie liegen zum einen in der Natur der Protagonisten: „Ziele, Probleme und Herausforderungen glichen sich, weil es Tiere waren, die produziert werden sollten. Ihre physiologischen und psychologischen Eigenschaften diktierten dem Produktionsprozess gewisse Spielregeln“ (141). Damit rückt Settele die landwirtschaftlichen Tiere als Lebewesen in den Fokus, beziehungsweise ihre Körper. Zum anderen sind die Ähnlichkeiten in den Ansprüchen begründet, die an die landwirtschaftliche Tierhaltung gestellt wurden: eine Steigerung der Produktion tierischer Lebensmittel pro Tier bei gleichzeitiger Reduzierung des menschlichen Arbeitseinsatzes. Und der Weg dorthin führte in beiden deutschen Staaten über die „Optimierung der Körper der Tiere, eine effiziente Unternehmensführung ihrer Haltung und die umfassende Technisierung der Ställe“ (316).

Aus diesem Dreiklang ergeben sich die drei Hauptkapitel der Arbeit, in deren Zentrum jeweils eine landwirtschaftliche Tierart steht. Im ersten Teil untersucht Settele die körperliche Optimierung der Rinder. Als Arbeitstiere immer weniger gebraucht, galten schließlich auch Rinder vor allem als Lebensmittel-Lieferanten. Zum Erhalt von mehr Milch und mehr Fleisch wurden die Alltagspraktiken Füttern und Melken ebenso in den Dienst genommen wie die Reproduktion. Während das Füttern „zu einem ausgedehnten Wissensregime“ (63) wurde, basierend auf der Popularisierung wissenschaftlicher Erkenntnisse zur optimalen Zusammensetzung und Verwertung des Futters, erlaubte die Mechanisierung des Melkens eine „neuartige Dimension menschlicher Arbeitsproduktivität“ (95). Und die Reproduktion der Rinder wurde schließlich auf Grundlage vielfach gesammelter Leistungsdaten systematisch kontrolliert und mittels künstlicher Besamung gesteuert.

Im zweiten Teil rekonstruiert Settele die konsequente Rentabilisierung am Beispiel der Hühnerhaltung. Sie wandelte sich besonders tiefgreifend von einem in der Verantwortung der Bäuerin stehenden Nebenerwerb zu einem kapitalintensiven, eigenständigen Wirtschaftszweig, in dem die körperlichen Prozesse der Tiere in Zahlen übersetzt und skalenökonomisch durchkalkuliert wurden. Entsprechend wuchs die Zahl der gehaltenen Tiere fast exponentiell. Sie wurden nun in platzsparenden Käfigen bei künstlichem Licht gehalten, mit Blick auf den „Veredelungskoeffizienten“ gefüttert, und ihr Leben wurde auf die produktivste Phase beschränkt. Mit den Hühnern richtet Settele ihren Blick auch auf die außerlandwirtschaftliche Gesellschaft, die (zumindest in der Bundesrepublik) zunehmend gegen die Haltungsbedingungen protestierte.

An den Schweinen zeigt Settele im dritten Teil schließlich, wie die Technisierung der Ställe zur Steigerung der Produktivität beitrug. Spaltenböden erlaubten nicht nur die Automatisierung des Ausmistens, sondern auch die Einsparung von Platz und Arbeit, und durch flexible Einrichtungen ließen sich Ställe an das Wachstum der Tiere anpassen und optimal ausnutzen. Zudem verhinderte die Fixierung von Muttersauen das Erdrücken von Ferkeln. Auch die Schweinehaltung rief gesellschaftliche Proteste hervor, die sich gegen die Belastungen von Luft und Boden durch die Ausscheidungen der Tiere richteten.

Während Veronika Settele für ihre Analyse einen klar abgegrenzten Raum und Zeitraum wählt und dabei die drei wichtigsten landwirtschaftlichen Tierarten an der Schnittstelle von „Agrar‑, Tier‑, Wirtschafts‑, Wissens- und Moralgeschichte“ (20) untersucht, nimmt Paul R. Josephson zwar eine breitere Raum‑/Zeit-Perspektive mit US-amerikanischem Schwerpunkt ein, konzentriert sich aber auf nur eine Tier- und Nutzungsart, nämlich das Masthuhn („broiler“). Seinen internationalen Aufstieg im 20. Jahrhundert versteht er als „political history, environmental history and history of technology“ (13).

Auch Josephson nimmt sich Ställe vor, und zwar die sogenannten CAFOs („Concentrated Animal Feeding Operation“). Diese Form der intensiven Tierhaltung, bei der beispielsweise bis zu 125.000 Masthühner in einer Einheit zusammengefasst sind, etablierte sich ab den 1920er Jahren zunächst in den USA und verbreitete sich schließlich weltweit. Mit „factory farms“ übernimmt Josephson den Begriff der Kritiker und betrachtet entsprechend das Masthuhn als „industrial object“ (3): „The broiler has been developed into a highly efficient meat-producing machine“ (6). Im so gesetzten Rahmen zeigt er auf, wie Lebewesen in globale industrielle Kontexte eingepasst wurden und werden, welche Probleme daraus für Tiere, Menschen und Umwelt resultier(t)en und welche internationalen Nuancen es politisch bedingt gab und gibt. „Unfettered capitalism“ sieht der Autor als Triebfeder für diese intensive Tierhaltung (8), verknüpft mit technologischem Fortschritt (17). Damit rücken Wirtschaftsform und Technik in den Fokus seiner Untersuchung, die er in sechs Kapitel gliedert.

Im ersten Kapitel beleuchtet Josephson die Rolle von Hühnern als kulturelle Repräsentationen, bei religiösen Handlungen oder in Wettbewerben und Wettkämpfen. Im zweiten Kapitel stellt er dar, wie sich der wissenschaftliche Blick auf das Huhn „from a natural to a technological foundation“ (23) gewandelt hat. Noch heute gültige ethologische und biologische Erkenntnisse vom Anfang des 20. Jahrhunderts stehen dabei im Kontrast zur Haltungspraxis in CAFOs, die wider besseren Wissens auch durch einen Schulterschluss von Industrie und Wissenschaft vorangetrieben wurde. Im dritten Kapitel zeichnet Josephson die Transformation des Huhns zum „industrial object“ nach. Durch zahlreiche Praktiken wie Zucht, Schnabelkürzen, technologisierte Fütterung, Unterbringung, Tötung, Verarbeitung und Vertrieb wurden die Tiere zu „meat machines“ (87) in einem auf mehrere Ebenen aufgeteilten Produktionsprozess gemacht, schnell und reichhaltig wachsend und nur durch die prophylaktische Gabe von Antibiotika vor Krankheiten und Seuchen bewahrt. Um die ‚Nebenwirkungen‘ dieser Transformation geht es im 4. Kapitel, in dem Josephson die verheerenden Auswirkungen der intensiven Haltung von Masthühnern auf die Umwelt verdeutlicht. Der anfallende Mist übersteigt den Bedarf der Landwirte an Dünger, er verschmutzt ebenso wie die Rückstände der Medikamente, die Kadaver verendeter Tiere, das Methan, die Plastikverpackungen und weiterer Abfall Boden, Wasser, Luft und Klima. Weitgehend fehlende staatliche Regulierungen verschärfen das Problem. Im 5. Kapitel stellt Josephson dar, wie sich der Protest gegen die CAFO-Haltung von Hühnern und anderen Tieren auf verschiedenen, moralischen, ökonomischen wie staatlich-regulativen Ebenen zu formieren begann, und zwar vornehmlich in offenen Gesellschaften. Schließlich verfolgt Josephson im 6. Kapitel den Weg der Masthühner als internationale Handelsware und stellt die Haltungs‑, Verbrauchs- und Handelspraktiken einiger führender Akteure wie Brasilien, China und Russland dar. Dabei beleuchtet er die negativen Auswirkungen des globalen Handels etwa auf lokale Strukturen der Hühnerhaltung oder auch auf die Gesundheit von Tieren und Menschen.

Die beiden Arbeiten gehen ihr Thema unterschiedlich an. Veronika Setteles Analyse beruht auf einem umfangreichen Quellenkorpus, sorgfältig erschlossen und eng in die Erzählung eingebunden. Das wirkt besonders anschaulich, lässt bisweilen aber auch nötige Distanzierungen beziehungsweise kontextuelle Einordnungen vermissen. Beispielsweise übernimmt Settele mitunter die in ihrem Quellenmaterial propagierte Einteilung in (wissenschaftliche) „Experten“ auf der einen Seite und „Praktikerinnen und Praktiker im Stall“ (64) auf der anderen und blendet die vielschichtigen Realitäten des landwirtschaftlichen Lebens und Arbeitens mit ihrer eigenen Form der Expertise aus. Paul R. Josephson dagegen verzichtet weitgehend auf Quellenanalysen und liefert eine auf Sekundärliteratur basierende enzyklopädische Übersicht, die weniger ausgewogen als tendenziös anmutet. Während Settele ihre Geschichte unaufgeregt, ohne explizite moralische Wertung erzählt und allein durch die Auswahl der Zitate kritische Akzente setzt, bezieht Josephson klar Stellung, die sich auch in seiner Wortwahl widerspiegelt: „chicken-gulag“ (2). Er plädiert zwar für eine Haltung, die den natürlichen Bedürfnissen von Hühnern gerecht wird, nimmt also die Tiere in den Blick – trotzdem bleiben sie bei ihm Objekte und ohne Einfluss in einem von Kapital, Wissenschaft und Technik bestimmten Prozess. Settele sieht die Tiere zugleich aber auch als Lebewesen, die durch ihre Körperlichkeit durchaus wirkmächtig sein können. Bei beiden Autoren erscheinen die Ausprägungen der intensiven Tierhaltung beinahe zwangsläufig oder alternativlos – hier wüsste man gerne mehr über die jeweiligen Wechselwirkungen und Verflechtungen in Forschung, Wirtschaft, Politik und Gesellschaft, die die Rahmenbedingungen für genau diese Entwicklungen darstellten.

Beide Arbeiten leisten auf verschiedenen Ebenen wertvolle Beiträge zur Geschichte der landwirtschaftlichen Tiere. Während Setteles empirisch gestützte Studie Leerstellen in den Geschichtswissenschaften füllt, liefert Josephson historisch fundierte Argumente für Politik und Moral. So ergänzen sich die Arbeiten und zeigen deutlich den Handlungsbedarf in der gegenwärtigen intensiven Tierhaltung auf.

